# Assessment of Knowledge, Attitude, and Practices of Indian Subpopulation on Third Molar Impaction, Etiology, and Management: A Questionnaire-Based Study

**DOI:** 10.7759/cureus.102878

**Published:** 2026-02-03

**Authors:** Janhavi Modi, Poorva S Sawant, Shifa S Satkut, Shreyas H Gupte, Shruti Singh, Drishti Shah

**Affiliations:** 1 Oral and Maxillofacial Surgery, Dr. G. D. Pol Foundation's YMT Dental College and Hospital, Navi Mumbai, IND; 2 Dentistry, Dr. G. D. Pol Foundation's YMT Dental College and Hospital, Navi Mumbai, IND

**Keywords:** attitude, awareness, impaction, knowledge, third molar

## Abstract

Introduction

Third molars are the most commonly impacted teeth in the oral cavity. Misconceptions among the general population regarding the complications associated with their removal have resulted in an aversion toward surgical extraction. To understand the current mindset among Indians, we aimed to assess the knowledge, attitude, and practices (KAP) of the Indian subpopulation toward third molar impactions, their associated complications, and management.

Materials and methods

A total of 716 participants from the outpatient department of a dental college completed a structured questionnaire-based cross-sectional study, which assessed KAP regarding third molar impaction, its etiology, and management. Data were collected via physical forms and Google Forms (Google, Inc., Mountain View, CA). Responses were coded numerically, and descriptive statistics (frequencies and percentages) were used to summarize KAP across demographic groups. No inferential statistical analysis was performed.

Results

A total of 716 participants completed the survey, including 518 females (72.3%) and 198 males (27.7%). In the knowledge domain, 93.2% (n = 667) correctly identified third molars as wisdom teeth, 57.8% (n = 414) knew the total number of third molars, 67.9% (n = 486) identified the usual eruption age, 71.2% (n = 510) were aware that third molars are the most commonly impacted teeth, and 43.3% (n = 310) correctly recognized lack of space as the main cause of impaction. In the attitude domain, 51.5% (n = 369) believed third molars do not play a significant role in mastication. Pain was cited as the most common problem by 65.6% (n = 470), while 21.1% (n = 151) perceived frequent associations with cysts or tumors. Regarding management, 41.1% (n = 294) preferred to wait for the onset of symptoms or complete eruption before extraction In the practice domain, 63.8% (n = 457) would consult a dentist for symptoms, 82.4% (n = 590) were willing to follow a dentist’s advice for extraction, and 41.8% (n = 299) expressed willingness to undergo minor surgical extraction under local anesthesia, while 58.2% (n = 417) were hesitant or uncertain. Support for educational initiatives, including audiovisual aids, was high at 94.0% (n = 673).

Conclusion

Thus, our study highlights the need for increased public awareness regarding the implications of third molar impaction and the importance of timely intervention. Greater emphasis on educational initiatives should be placed to inform the population about the potential risks of untreated impactions and the benefits of early management.

## Introduction

Third molars, widely known as wisdom teeth, represent the final stage of dental development and typically emerge between ages 17 and 25 [1]. These teeth can give rise to pathological conditions such as cysts, tumors, and pericoronitis [1,2-8]. These teeth are well known for their high propensity to become impacted. Impaction occurs when wisdom teeth fail to erupt into their functional position, remaining embedded in bone or soft tissue [1,4-5]. The most common cause for impaction in the mandible is insufficient space between the distal surface of the second molars and the anterior border of the ramus of the mandible; in the case of the maxilla, it is usually due to inadequate bony remodeling in the tuberosity region [1,5].

Certain complications are more frequently seen during surgical removal of mandibular third molars, including injury to the inferior alveolar nerve, periodontal compromise, infection, and alveolar osteitis (dry socket) [2-7,9].

There is stigma and fear among the general population regarding complications such as weakening of eyesight and difficulty in eating associated with the extraction of wisdom teeth. Widespread myths among the general population regarding these complications have resulted in an aversion to undergoing surgical extraction.

Asymptomatic third molars, which have the potential to develop cysts or tumors, are often perceived as harmless and therefore ignored. This shows a lack of awareness and a tendency to overlook the need for timely surgical intervention. Therefore, this study aims to evaluate the knowledge, attitudes, and practices (KAP) related to third molar impactions, through a validated, questionnaire-based survey, designed to evaluate understanding and perception of the condition amongst the Indian subpopulation.

## Materials and methods

This questionnaire-based cross-sectional study was conducted to assess the KAP related to third molar impaction, its etiology, and management among an Indian subpopulation. The study was carried out in the Department of Oral and Maxillofacial Surgery of a reputed dental college and hospital in India.

Study population and eligibility criteria

Individuals aged between 18 and 45 years from the general population who reported to the outpatient department during the study period were considered eligible for inclusion. Dental students and dental practitioners were excluded to avoid professional bias. Table 1 summarizes the inclusion and exclusion criteria.

**Table 1:** Inclusion and exclusion criteria

Inclusion Criteria	Exclusion Criteria
Individuals aged 18-45 years	Dental students
General population attending, OPD	Dental practitioners

Ethical approval

Ethical clearance for the study was obtained from the Institutional Ethics Committee (Approval No. YMTDC/IEC/OUT/2024/229-P). The questionnaire used in the study underwent expert validation for content and relevance prior to administration, and informed consent was obtained from all participants, with anonymity and confidentiality strictly maintained. Participation was voluntary, and written informed consent was obtained from all participants prior to data collection. Participants were assured of confidentiality and anonymity of the collected information.

Sample size estimation

The sample size was calculated using the estimates from the parent articles and using a single proportion formula, assuming an expected prevalence (p) of 40%, with 5% margin of error and a 95% confidence level:

$n = \frac{Z^{2} \times p \times (1 - p)}{d^{2}}$

where Z = 1.96 at 95% confidence level, p = 0.40, and d = 0.05.

The calculated minimum sample size was 368.7, which was rounded up to 370. To improve the reliability and representativeness of the findings, a total of 716 participants were included in the final analysis, exceeding the minimum required sample size by approximately 93.5% [1-5].

Data collection instrument

Data were collected using a structured, self-administered questionnaire comprising three domains: KAP (see Appendices).

The knowledge domain consisted of five closed-ended questions assessing awareness of third molars, their eruption age, number, impaction, and etiology. The attitude domain included four close-ended questions evaluating perceptions regarding the functional role of third molars, associated problems, potential pathologies, and management preferences. The practice domain comprised three close-ended questions exploring participants’ responses to third molar-related symptoms, acceptance of professional advice, and willingness to undergo surgical intervention.

Data management and statistical analysis

Completed questionnaires obtained through both physical forms and Google Forms (Google, Inc., Mountain View, CA) were reviewed for completeness and consistency. Responses were coded and entered into a database for analysis. Descriptive statistics were used to summarize the data. Categorical variables were expressed as frequencies and percentages and are presented in tables and textual form. No inferential statistical analysis was performed, as the primary objective of the study was to descriptively assess the levels of KAP related to third molars among the study population.

## Results

A total of 716 individuals participated in the survey, exceeding the calculated minimum sample size. The majority of respondents were female (n = 518, 72.3%), while males constituted 27.7% (n = 198). The findings related to KAP regarding third molar impaction are presented as frequencies and percentages of the responses received.

Knowledge domain

Most participants demonstrated basic awareness regarding third molars (Table 2). The majority (93.2%, n = 667) correctly identified third molars as wisdom teeth, while 6.8% were unaware of this terminology. Regarding the number of third molars, 57.8% (n = 414) correctly reported the presence of four third molars, whereas 42.2% provided incorrect responses or reported uncertainty.

**Table 2:** Distribution of responses in the knowledge domain

Sr. No.	Questions	Options Provided	Correct Response	Percentage of Population Given Correct Response	Percentage of Population Given Incorrect Response
1	Are you aware that third molars are called wisdom teeth?	a) Yes. b) No. c) Don’t know.	Yes	93.2% (n = 667)	6.8%
2	Do you know how many third molars are present in the mouth?	a) 1. b) 2. c) 3. d) 4. e) Don’t know.	4	57.8% (n = 414)	42.2%
3	Do you have any idea at what age do third molars commonly erupts?	a) 12-15 years. b) 17-21 years. c) 25 years and above. d) Don’t know.	17-25 years	67.9% (n = 486)	32.1%
4	Do you know that the third molars are the most commonly impacted teeth?	a) Yes. b) No. c) Don’t know.	Yes	71.2% (n = 510)	28.8%
5	Do you know the cause for impaction of third molars?	a) No space to accommodate. b) History of trauma (fall accident). c) Because it’s the last tooth to erupt. d) Don’t know.	No space to accommodate	43.3% (n = 310)	56.7%

With respect to eruption age, 67.9% (n = 486) correctly identified the typical eruption period as 17-25 years, while 32.1% were unaware or answered incorrectly. Awareness that third molars are the most commonly impacted teeth was reported by 71.2% (n = 510) of respondents, while 28.8% lacked this knowledge.

Knowledge regarding the etiology of third molar impaction was comparatively limited among participants. Only 43.3% (n = 310) correctly identified lack of space as the primary cause of impaction, whereas 56.7% gave incorrect responses or reported uncertainty.

Attitude domain

Attitudes toward the function and management of third molars varied among participants (Table 3). Slightly more than half (51.5%, n = 369) reported that third molars do not play a significant role in mastication, while 48.5% believed they do or were unsure.

**Table 3:** Distribution of responses in the attitude domain

Sr. No.	Questions	Options Provided	Correct Response	Percentage of Population Given Correct Response	Percentage of Population Given Incorrect Response
6)	Do you think that third molars play any role in chewing?	a) Yes. b) No. c) Don’t know.	No	51.5% (n = 369)	48.5%
7)	What problems do you think third molars can cause?	a) Pain. b) Difficulty in opening mouth. c) Swelling. d) Decay to other teeth. e) Don’t know.	Pain	65.6% (n = 470)	34.5%
8)	How often do you think impacted molars are associated with some abnormality like cyst and tumors?	a) Very often. b) Often. c) Rarely. d) Never. e) Don’t know.	Often	21.1% (n =151)	78.9%
9)	What measures will you take if you come to know that your third molars are impacted?	a) Get it extracted sooner. b) Wait for it to completely erupt or show any symptoms then get it extracted. c) Never get it extracted.	Wait for it to completely erupt or show any symptoms then get it extracted	41.1% (n = 294)	58.9%

Pain was identified as the most common problem associated with third molars by 65.6% (n = 470) of respondents, whereas 34.5% selected other options or were uncertain. When asked about the association of impacted third molars with cysts or tumors, only 21.1% (n = 151) believed such associations are common; 78.9% perceived these complications as rare, nonexistent, or were unsure.

Regarding management preferences, 41.1% (n = 294) reported that they would wait for complete eruption or the onset of symptoms before considering extraction. The remaining respondents preferred early extraction or expressed reluctance toward extraction.

Practice domain

Practice-related responses are summarized in Table 4. In the event of wisdom tooth pain, 63.8% (n = 457) reported that they would consult a dentist as the first step, while 36.2% opted for alternative measures such as ignoring the pain, using home remedies, or self-medication.

**Table 4:** Distribution of responses in the practice domain

Sr. No.	Questions	Options Provided	Correct Response	Percentage of Population Given Correct Response	Percentage of Population Given Incorrect Response
10)	What is the first step you will take if your wisdom tooth starts paining?	a) Ignore the pain. b) Do some home remedies like applying clove oil, salt water rinse cold compression etc. c) Go to pharmacy and take medicines. d) Visit the dentist.	Visit the dentist	63.8% (n = 457)	36.2%
11)	If your dentist ask you to extract your impacted third molar will you do it?	a) Yes. b) No.	Yes	82.4% (n = 590)	17.6%
12)	Will you accept a minor surgery (on chair) under local anesthesia as an option for the extraction of impacted third molars?	a) Yes. b) No. c) Maybe. d) Don’t know.	Yes	41.8% (n = 299)	58.2%
13)	Do you think we should make ads or videos to educate people about wisdom teeth?	a) Yes. b) No.	Yes	94% (n = 673)	6%

Most participants (82.4%, n = 590) indicated that they would agree to extraction if advised by a dentist; however, 17.6% were unwilling to undergo extraction despite professional consultation. Willingness to undergo minor surgical extraction under local anesthesia was reported by 41.8% (n = 299), whereas 58.2% expressed hesitation, refusal, or lack of awareness regarding the procedure.

Support for public awareness initiatives was high, with 94% (n = 673) endorsing the use of advertisements or educational videos to improve awareness about wisdom teeth; 6% felt such initiatives were unnecessary.

## Discussion

Population

Third molar impactions occur due to inadequate space in the retromolar region. Due to the late eruption, third molars and their unique position in the arch pose many complications and thus require timely attention for extraction.

According to Muhsin et al., the majority of dental extractions, accounting for 18%, involve the lower third molars, making them the most commonly removed teeth [9].

Alesia et al. reported that third molars in the lower jaw (19.4%) and upper jaw (16.4%) were the most commonly extracted teeth [10].

According to Jafarian et al., lower third molars accounted for the highest percentage of extractions, constituting 18% (473) of all extracted teeth [11].

In our study, 716 participants have given their responses, of whom 590 participants are willing to undergo third molar extraction, while 126 participants are hesitant to proceed with the procedure to remove their impacted third molars.

When participants were asked about acceptance of minor surgical extraction under local anesthesia, 299 respondents agreed to undergo the procedure, while the remaining participants either declined or expressed uncertainty.

Causes

Alfadil et al. categorized third molar extractions into two groups based on the presence or absence of symptoms. Symptomatic third molars were defined as those that caused pain, disrupted chewing function, or were associated with cysts or tumor lesions. On the other hand, asymptomatic third molars were those that did not exhibit any noticeable issues or symptoms, presenting no apparent problems or complications [12].

While in our study, participants perceived pain as the primary indication for third molar extraction, with a significant 470 individuals citing this as the primary cause.

According to Dodson et al., other reasons include incisor crowding and a negative impact on work or education. Additionally, extraction may be necessary due to oral health concerns, damage to adjacent teeth or restorations, maxillofacial lesions like odontogenic cysts or tumors, facial cellulitis, or the potential need for future extraction of initially asymptomatic wisdom teeth [13].

According to our survey, swelling was the second most common reason for extraction, reported by 78 participants. Other reasons included prevention of caries to adjacent teeth (68 participants) and difficulty in mouth opening (39 participants). However, 61 participants were uncertain about the reason for third molar extractions.

Audiovisual aids 

According to Choi et al., audiovisual education provided before surgery can increase patient knowledge and reduce anxiety following impacted wisdom tooth removal. It also showed that patients who were informed through audiovisual means experienced a notable reduction in anxiety levels one week post-surgery, scoring significantly lower than the control group [14].

According to Sainchar et al., watching surgical videos can educate patients, but may also increase anxiety due to graphic content. A study by Kazancioglu et al. found that patients who watched videos before oral surgery experienced higher anxiety levels. Visual exposure doesn't always lead to better preparedness. Patient anxiety levels should be considered when using videos as educational tools [15].

According to Izham Akmal et al., audiovisual aids prepare patients for dental procedures, reducing anxiety and increasing comfort. They offer an effective alternative to sedation and promote oral health through self-directed education. This approach can increase awareness and reduce preventable oral diseases [16].

Our findings are in agreement with existing literature, further reinforcing the importance of patient education in addressing hesitancy toward surgical extraction. A significant majority of participants, 673 out of 716, believed that educating the general population through visual aids would help alleviate their concerns and increase acceptance of surgical extractions. Despite the desire for knowledge, there is a significant gap in accessible educational resources. We advocate for a collaborative effort between the dental community, educational institutions (such as schools and colleges), and non-governmental organizations (NGOs) to develop and disseminate informative materials to educate the general population, and also those from rural areas and with lower educational backgrounds, to make informed decisions about surgical extractions.

Fears and stigma

Research by Suleiman et al. revealed that female patients are over four times more likely to experience anxiety than male patients, and individuals who have undergone previous surgical tooth extractions are nearly four times more likely to experience anxiety compared to those without prior extraction experience [17].

While previous studies report higher anxiety levels among female patients, our findings indicate greater willingness among female participants to seek management for impacted third molars, whereas men may require additional motivation and encouragement to consider surgical intervention.

A study by Jeddy et al. investigated the underlying causes of dental fear and anxiety-inducing procedures. The results showed that previous negative dental experiences were the primary trigger for dental fear in approximately one-third (33.3%) of patients. Furthermore, pain was identified as the main source of anxiety for nearly 80% (79.7%) of patients. When it came to specific dental procedures, extractions were found to cause the most anxiety (46.4%), closely followed by root canal treatments, highlighting the need for gentle and compassionate dental care to alleviate patient concerns [18].

According to a study by Niemczyk et al., the physical and psychological impact of the procedure can cause distress, with common concerns including fear of pain or discomfort, perioperative stress, and worry about potential complications. Notably, the study found that young women and individuals from rural areas experience higher levels of anxiety, highlighting the need for tailored approaches to address these concerns and provide supportive care during dental surgery. By understanding the sources of anxiety, dentists can take steps to mitigate patients' fears and make the experience more comfortable and stress-free [19].

Our research reveals a notable gender difference in attitudes toward the surgical extraction of impacted third molars. Females demonstrated a higher level of awareness and willingness to undergo surgical extraction compared to their male counterparts.

Qualification

According to Mohite et al., a study surveyed 183 patients (98 men and 85 women) about common myths surrounding wisdom teeth. The patients were divided into two groups: educated and less educated. The results showed that many patients held misconceptions, such as believing wisdom tooth removal can cause eye problems, sneezing issues, facial deformity, or even cancer. However, a significant number of patients were also aware of the facts, knowing that wisdom tooth surgery does not typically lead to these issues. The study highlights the need for better education and awareness about wisdom teeth and their treatment [20]. 

According to our survey, 37 respondents expressed concern that the extraction of their third molar (wisdom tooth) could lead to weakened eyesight, citing this as their reason for not being ready to undergo the procedure. This finding suggests that reluctance toward extraction may be influenced by misconceptions and traditional beliefs, underscoring the need for targeted educational interventions.

Traditional methods

Singh et al. reported that, in an attempt to avoid dental visits, patients often resort to traditional remedies and local beliefs to self-treat their oral health issues. They may turn to herbs, alum mouth rinses, and other natural products in search of relief. It is only when these local remedies fail to deliver that patients seek professional help at a nearby clinic. However, this reliance on unproven methods can lead to delayed diagnosis and treatment [21-22].

According to our study, when respondents were asked about the first step they would take when their wisdom tooth starts paining, majority (457 respondents) chose the option, to visit the dentist followed by (180 respondents) trying home remedies such as clove oil, saltwater rinse, or cold compression Some individuals preferred to purchase over-the-counter medication at a pharmacy (46 respondents), while a small group chose to ignore the pain (33 respondents). Although most participants reported visiting a dentist as the first step, a considerable proportion initially relied on home remedies or self-medication, which may delay appropriate dental care.

A key limitation of our study is the gender imbalance, with a disproportionately higher representation of female participants, potentially introducing bias into the findings on gender-specific awareness. Furthermore, the study's institutional setting constrains the participant pool, limiting the generalizability of the results to the wider population. Despite these limitations, the findings underscore the need for targeted patient education and counseling in clinical dental practice to improve awareness regarding third molar impaction and its management. Future studies should include multicentric designs with balanced demographic representation and standardized assessment tools to enhance external validity and strengthen evidence applicability.

## Conclusions

Despite basic awareness of third molar impactions among the general population, significant gaps remain in understanding their potential complications and the need for surgical management. Limited knowledge about the possible consequences of untreated impacted third molars may lead to hesitation in seeking timely care.

Our study highlights the importance of raising public awareness and fostering informed decision-making about the management of third molars. Educational awareness in the form of targeted campaigns, audiovisual aids, and community-based programs can help in bridging these gaps by highlighting the benefits of early intervention. Overall, the findings of this study provide valuable insights into the understanding of the general population, their attitudes and practices toward the management of impacted third molars, underlining the need to promote awareness to support prompt management.

## Appendices

Questionnaire

Name (optional):

Age :

Sex:

·      Male     

·      Female      

Qualification:

a) Schooling (10th grade)

b) 12th grade

c) Graduation

d) Post-graduation

e) Others

Knowledge

1)    Are you aware that third molars are called wisdom teeth?

o   Yes

o   No

o   Don’t know

2)    Do you know how many third molars are present in the mouth?

o   1

o   2

o   3

o   4

o   Don’t know

3)    Do you have any idea at what age third molars commonly erupt?

o   12-15 years

o   17-21 years

o   25 years and above

o   Don’t know       

4)    Do you know that the third molars are the most commonly impacted teeth?

o   Yes

o   No

o   Don’t know

5)    Do you know the cause of impaction of third molars

o   No space to accommodate

o   History of trauma (fall, accident)

o   ⁠Because it’s the last tooth to erupt

o   ⁠Don’t know

Attitude

6)    Do you think that third molars play any role in chewing?

o   Yes

o   No

o   Don’t know

7)    What problems do you think third molars can cause?

o   Pain

o   Difficulty in opening the mouth

o   Swelling

o   Decay to other teeth

o   Don’t know 

8)    How often do you think impacted molars are associated with some abnormality like cysts and tumors (large swelling)?

o   Very often

o   Rarely

o   Never

o   Don’t know

9)    What measures will you take if you come to know that your third molars are impacted?

o   Get it extracted sooner

o   Wait for it to completely erupt or show any symptoms, then get it extracted

o   Never get it extracted

o   Don’t know

Practices

10) What is the first step you will take if your wisdom tooth starts aching?

o   Ignore the pain

o   Do some home remedies like applying clove oil, salt water rinse, cold compression, etc.

o   Go to the pharmacy and take medicines

o   Visit the dentist.

11) If your dentist asks you to extract your impacted third molar, will you do it?

o   Yes

o   No

a)     If yes, why?

o   Because it can cause problems (pain, swelling in the future)

o   Because it can cause decay in other teeth

o   Because the dentist said so

b)    If no, why?

o   Because it is asymptomatic

o   Because you believe that getting a tooth extracted leads to the weakening of the eyesight

o   ⁠Because you don’t want to go for unnecessary surgical intervention

o   ⁠You will get it treated when it starts showing symptoms

12) Will you accept a minor surgery (on chair) under local anesthesia as an option for the extraction of impacted third molars?

o   Yes

o   No

o   Maybe

o   Don’t know

13)  Do you think we should make ads or videos to educate people about wisdom teeth?

o   Yes

o   No
